# Multiple Tubercular Cervical, Supraclavicular, and Pretracheal Lymphadenitis With Scrofuloderma: A Rare Case

**DOI:** 10.7759/cureus.51134

**Published:** 2023-12-26

**Authors:** Sankalp Yadav

**Affiliations:** 1 Medicine, Shri Madan Lal Khurana Chest Clinic, New Delhi, IND

**Keywords:** cbnaat/xpert/rif assay, fine needle aspiration cytology (fnac), cervical tuberculous lymphadenitis, scrofula, scrofuloderma

## Abstract

In clinical settings, cutaneous tuberculosis is an uncommon occurrence. On the other hand, incidences of this kind of tuberculosis are usually documented in high-burden nations. Despite being the most prevalent variety of cutaneous tuberculosis, scrofuloderma frequently goes undiagnosed. The present case is a rare case of simultaneous involvement of cervical, supraclavicular, and pretracheal lymph nodes and skin due to *Mycobacterium tuberculosis*. A 26-year-old Indian female was diagnosed after a careful diagnostic workup involving fine needle aspiration cytology, a cartridge-based nucleic acid amplification test, and a biopsy. The case is remarkable as there was no pulmonary involvement. She was initiated on antitubercular treatment per national policy.

## Introduction

*Mycobacterium tuberculosis* can present as pulmonary and/or extrapulmonary tuberculosis [1]. Extrapulmonary tuberculosis is relatively rare (8.4-13.7%) [2]. Further, the common sites are lymph nodes, a pleura, the abdomen, bones and joints, the liver, the spleen, meninges, cutaneous, etc. [1]. On a comparative basis, cutaneous tuberculosis is an infrequent entity and represents just 1-1.5% of all extrapulmonary presentations [3].

All types of cutaneous tuberculosis can be caused by *M. tuberculosis*, *Mycobacterium bovis*, and the attenuated bacille Calmette-Guerin organisms [4]. Scrofuloderma, lupus vulgaris, tubercular verrucosa cutis, artificial cutaneous tuberculosis, tuberculous gumma, acute miliary cutaneous tuberculosis, and tuberculous chancre are among the several clinical manifestations of cutaneous tuberculosis [5]. However, scrofuloderma is the most common subtype of cutaneous tuberculosis [6].

As stated, extrapulmonary tuberculosis most commonly manifests as tubercular lymphadenitis. Another name for it is "scrofula." Known as "Kings Evil" in Europe, the illness was thought to be cured by the royal touch up to the 18th century [7]. In 60-90% of patients, cervical lymph nodes are the most prevalent location of tuberculous lymphadenopathy [8].

A very rare case of simultaneous involvement of scrofuloderma along with multiple cervical, supraclavicular, and pretracheal lymphadenitis is presented here. A detailed clinical examination, backed by thorough laboratory investigations, led to the final diagnosis. She was put on antitubercular chemotherapy per her weight.

## Case presentation

A 26-year-old female of Indian origin belonging to low socioeconomic status presented to the outpatient department with a history of multiple painful swellings in the right side and midline of the neck that had been gradually increasing in size for the past three months. She consulted local healers (unregistered medical practitioners) for the same, but there was no relief.

She was apparently alright three months ago when she had a fever, which was an evening rise not associated with chills or rigor. This was followed by a painless swelling (about 1 cm x 2 cm) in the right cervical region, which was not associated with discharge. Gradually, over the next 15 days, she developed multiple swellings in the right cervical, supraclavicular, and midline of the neck region. These swellings were associated with pain, and two of them were associated with a purulent, non-foul-smelling, yellowish-colored discharge. Moreover, there was a swollen 5 cm x 6 cm anterior chest wall lesion with ill-defined borders.

She was a migrant laborer and had no known medical issues, and her family and psychosocial history were unremarkable. Besides, there was no prior history of trauma, tuberculosis, or skin-related disorders. Moreover, there was no history of imprisonment or stay at refugee or night shelters.

A general examination revealed a lean female with these vitals: temperature of 98.4 degrees Fahrenheit, respiratory rate of 15/minute, blood pressure 110/80 mmHg, and SpO_2_ 99% on room air. There was no icterus, pallor, cyanosis, edema, clubbing, or koilonychia present.

**Figure 1:**
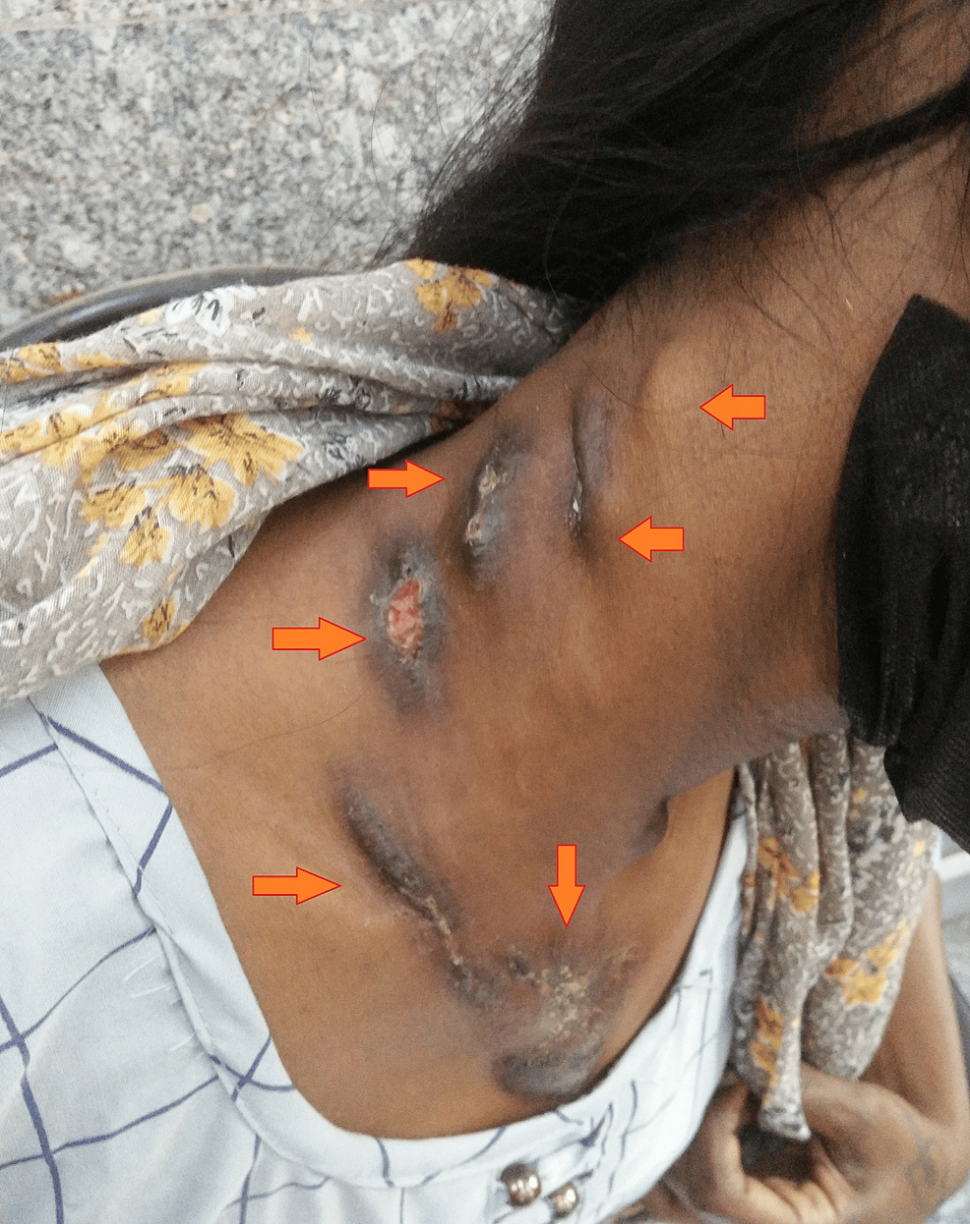
Gross image showing multiple cervical, supraclavicular, and anterior chest wall swellings

Her systemic examination was unremarkable. Dermatological examination revealed four swellings in the right cervical region, with sizes of 1 cm x 4 cm firm on consistency painless without discharging sinus, 5 cm x 2 cm with a discharging sinus, 4 cm x 2 cm ulcerated with two discharging sinuses, and 5 cm x 3 cm ulcerated with two discharging sinuses. Furthermore, there was a 3 cm x 1 cm swelling in the right supraclavicular region without discharging the sinus (Figure 1).

**Figure 2:**
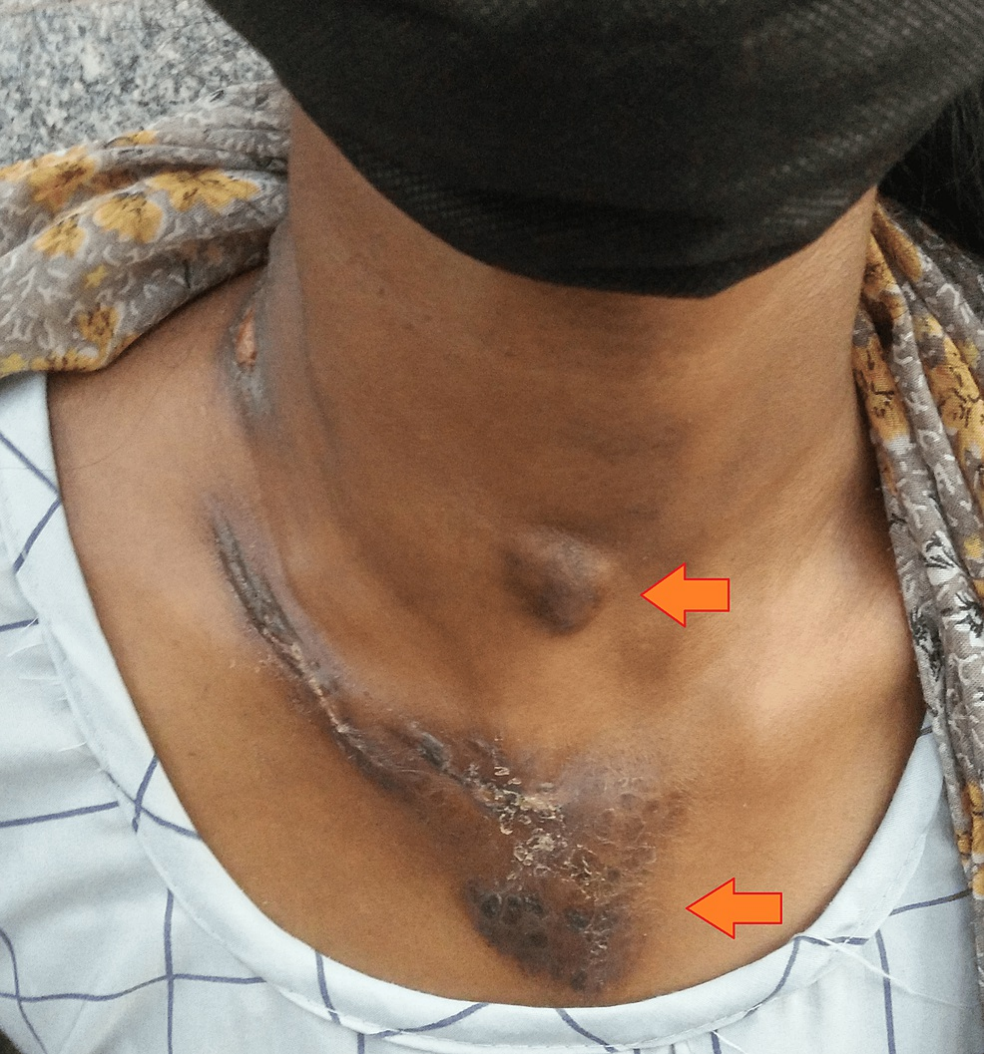
Gross image showing pretracheal lymph nodes and an anterior chest wall lesion

Additionally, there was a 2 cm x 3 cm mobile and painless pretracheal swelling without discharging the sinus in the neck. Furthermore, there was a painless, swollen 5 cm x 6 cm anterior chest wall lesion with ill-defined borders surrounded by a bluish and edematous skin surface about 1 cm from the jugular notch (Figure 2).

**Figure 3:**
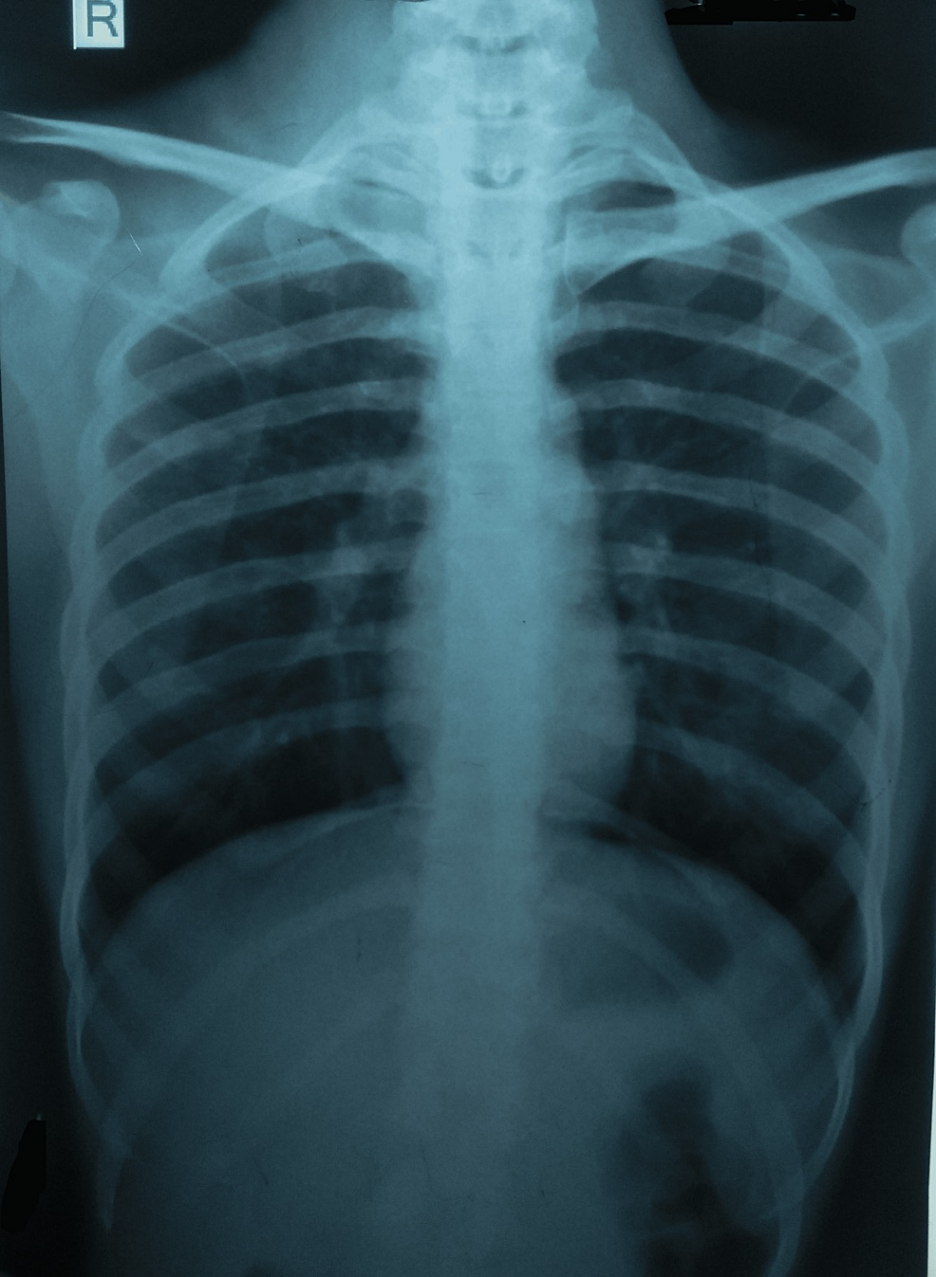
A normal chest radiograph

Besides, there were no engorged veins or similar findings in the left cervical or supraclavicular regions. Moreover, there were no other palpable nodes found in the submandibular, axillary, or submental stations. A diagnostic workup showed a normal chest radiograph (Figure 3).

Lab data showed a hemoglobin of 11.1 g/dl and an elevated erythrocyte sedimentation rate of 67 mm in the first hour. Her tuberculin skin test was positive with 18 mm indurations. All other laboratory parameters, including hepatitis A, B, and C, as well as HIV I and II, were within the normal range. Her induced sputum microscopy for acid-fast bacilli and cartridge-based nucleic acid amplification tests were negative. She underwent fine-needle aspiration cytology of the right cervical, right supraclavicular, and pretracheal swellings. The findings pointed to a few granulomas with a background of patchy necrosis that were composed of epitheloid cells, lymphocytes, and, at times, Langhans giant cells. Acid-fast bacilli were detected by Ziehl-Neelsen staining. The pus samples obtained were also sent for a cartridge-based nucleic acid amplification test, a line-probe assay, and an automated liquid culture. There was a detection of *Mycobacterium tuberculosis* on a cartridge-based nucleic acid amplification test (no resistance to rifampicin); however, the results of the line-probe assay and culture were negative.

Further, with a suspicion of scrofuloderma, a punch biopsy of the skin from an anterior chest wall lesion revealed atrophied epidermal lining, islands of trapped epidermis in the dermis, and lost rete pegs. There was a persistent inflammatory infiltrate underneath the dermis, primarily composed of lymphocytes and a small number of polymorphs with multinucleated giant cells here and there. There were no visible regions of edema, bleeding, congestion, or necrosis. Histopathological characteristics pointed to scrofuloderma or cutaneous tuberculosis.

Finally, a diagnosis of extrapulmonary tuberculosis of the right cervical, supraclavicular, and pretracheal lymphadenitis with cutaneous tuberculosis (scrofuloderma) was made, and medical management was initiated with fixed-dose combinations of isoniazid, pyrazinamide, rifampicin, and ethambutol every morning for eight weeks, constituting the intensive phase, followed by a 16-week continuation phase of isoniazid, rifampicin, and ethambutol.

There was a visible improvement in her swellings at the second-month follow-up, with the healing of sinuses, shrinking of swellings, and stoppage of the purulent discharge. She was advised incision and drainage at the treatment initiation per the latest national guidelines; however, she refused the same and was continued on antitubercular treatment. Moreover, advanced radiometric investigations were not done in this case because of financial constraints, a lack of free slots for the tests, and patient reluctance. Anyway, a diagnosis was already established with the fine-needle aspiration cytology, a cartridge-based nucleic acid amplification test of the involved lymph nodes, and a punch biopsy of the anterior chest wall skin lesion. After completing her treatment for four months, she was lost to follow-up.

## Discussion

Cutaneous tuberculosis was first described in a paper in 1981. Cutaneous tuberculosis shares many similarities with other skin lesions and cutaneous clinical manifestations; as a result, diagnosing it can be difficult for clinicians. The name "scrofula" was sometimes used to describe cervical tuberculous lymphadenopathy, a tuberculosis infection of the neck lymph nodes. Usually, a primary tuberculosis infection of the lymph node is the cause of it. Bacilli of *M. tuberculosis *can spread hematogenously and through lymph nodes [9].

Tuberculosis cutis colliquativa, also known as scrofuloderma, is the most prevalent type of cutaneous tuberculosis, especially in children. A bluish-red nodule covering an infected lymph node, a bone, or a joint is called scrofuloderma. The nodule eventually breaks down into an undermined ulcer with granulating tissue at its base. As the illness becomes more severe, irregular adherent lumps form that are fluctuant and associated with discharge at times and thickly fibrous at other times [9].

The diagnosis of scrofuloderma is challenging due to the non-specific nature of the lesions of the disease, ambiguity with other cutaneous manifestations, delayed presentations, the unavailability of positive culture results, a low index of suspicion, and a lack of awareness among the treating physicians [10].

Scrofuloderma is caused by cutaneous infection next to a tuberculous focus, which can be related to either bone, joint, or testicular tuberculosis or peripheral ganglia tuberculosis, which is the most frequent extrapulmonary form of tuberculosis in HIV-positive individuals and kids. Subcutaneous, painless, slowly growing nodules that develop into ulcers and fistulous tracts with serous, purulent, or caseous fluid draining are the hallmarks of the clinical presentation [11]. The progression is insidious and manifests as atrophic sequelae, chronic ulcers, persistent purulent drainage, or spontaneous healing. The most commonly challenged lymph nodes are the cervical ones, but the inguinal, axillary, pre- and post-auricular, submandibular, epitrochlear, and occipital lymph nodes may also be involved [12].

Actinomycosis, hidradenitis suppurativa, sporotrichosis, atypical mycobacteriosis, gummatous syphilis, and bacterial abscesses are among the conditions included in the differential diagnosis. The tuberculin skin test is typically very reactive, and the oldest lesions may be paucibacillary despite being historically categorized as a multibacillary variety. The histopathological observations reveal the presence of acid-fast bacilli and granulomatous inflammatory infiltration linked to caseous necrosis [13].

Chemotherapy is still the preferred course of treatment for both tubercular lymphadenitis and scrofuloderma. The goal is to expeditiously cure the illness to avert relapses and the formation of resistant strains. The same was followed in the present case with an initiation phase of two months with isoniazid, pyrazinamide, ethambutol, and rifampicin, followed by a four-month continuation phase with isoniazid, ethambutol, and rifampin [10]. For large lymph nodes, incision and drainage are indicated, along with six months of antitubercular medicines.

A case similar to the present case was reported by Soeroso et al., but the present case was unique in the age, gender, and involvement of pretracheal lymph nodes, in addition to the right cervical and supraclavicular lymph nodes, along with scrofuloderma [9]. Additionally, there was no involvement of post-auricular and axillary lymph nodes in the present case.

## Conclusions

A very unusual case of numerous cervical, supraclavicular, and pretracheal lymphadenitis occurring concurrently with scrofuloderma in an Indian female was reported. The definitive diagnosis was reached after a comprehensive laboratory study and a rigorous clinical assessment. Based on her weight, she was started on antitubercular chemotherapy. This case emphasizes the high degree of suspicion to be kept in mind while dealing with multiple swellings with discharging sinuses. Moreover, a delay could result in unfavorable outcomes.
